# Angiopoietin-1 Upregulates De Novo Expression of Il-1β and Il1-Ra, and the Exclusive Release of Il1-Ra from Human Neutrophils

**DOI:** 10.1371/journal.pone.0088980

**Published:** 2014-02-12

**Authors:** Lydia E. Haddad, Martin G. Sirois

**Affiliations:** 1 Research Center, Montreal Heart Institute, Montréal, Canada; 2 Department Of Pharmacology, Faculty of Medicine, Université De Montréal, Montréal, Canada; University of Tübingen, Germany

## Abstract

The expression of the angiopoietin (Ang) receptor, Tie2, on both endothelial and inflammatory cells supports the idea that Ang signaling may play a fundamental role in initiating and maintaining the inflammatory response. We have previously shown that Ang1 and/or Ang2 alter the innate immune response by enhancing human neutrophil survival, chemotaxis and production of inflammatory cytokine interleukin-8 (IL-8) *in vitro*. Thus, we hypothesized that Ang1 and Ang2 could modulate other inflammatory signals in neutrophils, a possibility we explored through a gene-based assay looking at changes in the mRNA expression of 84 inflammatory cytokines and their receptors. We observed that Ang1 (10^−8^ M), but not Ang2, increased mRNA expression of prominent pro-inflammatory cytokine IL-1β and its natural antagonist IL-1RA, by up to 32.6- and 10.0-fold respectively, compared to PBS-control. The effects of Ang1 extended to the proteins, as Ang1 increased intracellular levels of precursor and mature IL-1β, and extracellular levels of IL-1RA proteins, by up to 4.2-, 5.0- and 4.4-fold respectively, compared to PBS-control. Interestingly, Ang1 failed at inducing IL-1β protein release or at increasing intracellular IL-1RA, but the ratio of IL-1RA to mature IL-1β remained above 100-fold molar excess inside and outside the cells. The above-noted effects of Ang1 were mediated by MAP kinases, whereby inhibiting MEK1/2 lead to up to 70% effect reduction, whereas the blockade of p38MAPK activity doubled Ang1's effect. These findings suggest that Ang1 selectively alters the balance of neutrophil-derived inflammatory cytokines, favoring the blockade of IL-1 activity, a consideration for future therapies of inflammatory diseases.

## Introduction

Inflammation is characterized by a sequence of events that involve activation of the endothelium, release of endothelial mediators, vascular remodeling to allow for increased permeability and blood flow, and leukocyte – especially neutrophil – recruitment and infiltration into inflamed tissues. Because acute inflammation and angiogenesis can be triggered by the same molecular events, it is not surprising that most molecules that alter permeability, such as vascular endothelial growth factor (VEGF), tumor necrosis factor (TNF)-α and nitric oxide (NO), are potent pro-angiogenic factors (review; [1]).

Angiopoietins (Ang) are a family of angiogenic growth factors that play a major role in modulating vascular integrity and maturation. While the expression of the Ang receptor Tie2 on both endothelial and inflammatory cells [2]–[4] suggests a potential involvement in inflammation, a literature review of the specific contributions of the primary family members, Ang1 and Ang2, reveals a dichotomy of pro- and anti-inflammatory properties that is often influenced by the presence of other inflammatory mediators. From an anti-inflammatory perspective, Ang1 counteracts some components of the activity of pro-inflammatory factors on endothelial cells (ECs), inhibiting increases in EC permeability induced by VEGF, thrombin, bradykinin and histamine *in vitro* [5]–[7]. Additionally, Ang1 downregulates the release of chemokine IL-8 by ECs [6], and inhibits adherence and transmigration of neutrophils across EC monolayers stimulated with VEGF, TNF-α and thrombin [5], [6], [8], likely through altering the expression of endothelial E-selectin and intracellular/vascular cell adhesion molecules (ICAM-1/VCAM-1) [5], [9]. In mouse skin *in vivo*, co-overexpression of VEGF and Ang1 shows an additive effect on angiogenesis but results in leakage-resistant vessels with little inflammation [10]. In stark contrast, Ang1 exerts certain pro-inflammatory activities: Ang1 through Tie2 activation increases endothelial P-selectin translocation, a protein that mediates the rolling of leukocytes onto the endothelium under inflammatory conditions[11]. Ang1 has also the ability to directly impact leukocyte behavior, stimulating neutrophil IL-8 synthesis and release [12], and acting in a Tie2-dependent manner to recruit neutrophils and eosinophils, to increase neutrophil lifespan, and to promote neutrophil adhesion onto extracellular matrix [3], [4], [13]–[15]. The contribution of Ang2 to acute inflammation is even less defined, with some evidence of pro-inflammatory properties such as enhancing TNF-α-dependent adhesion of leukocytes to EC monolayers, as well as TNF-α-induced expression of ICAM-1 and VCAM-1 [16]. Ang2 alone also promotes a transient endothelial P-selectin translocation and its effects on neutrophil adhesion and chemoattraction are Tie2-dependent and similar to those of Ang1 [4], [15]; however, unlike Ang1, Ang2 fails to promote neutrophil IL-8 synthesis and/or release, to increase neutrophil survival, or to counteract the effects of Ang1 on the aforementioned processes [12], [13]. Thus, the distinct contributions of Ang1 and Ang2 to acute inflammation remain to be clearly delineated. Neutrophils are generally the first responders at sites of inflammation. They contribute substantially to inflammation through their ability to produce proteases, reactive oxygen species [17], [18], and to a lesser extent, cytokines including interleukin (IL)-6, TNF-α and IL-1 receptor antagonist (IL-1RA) [19]–[23]. Building on our recent findings that Ang1 promotes significant IL-8 production in human neutrophils *in vitro* in a time-dependent manner [12], we broadened our investigation to 84 other pro-inflammatory cytokines and their receptors, and looked at changes in their mRNA expression following angiopoietins stimulation. The first part of this study identified three related targets, all belonging to the IL-1 family of inflammatory cytokines, IL-1α, IL-1β, and IL-1RA, as well as a number of other potential interests unrelated to the IL-1 family. The second part of this study focused on identifying the kinetics and mechanisms that mediate the effects of Ang1 on IL-1 family members in neutrophils.

## Materials and Methods

### Neutrophil purification

The study was conducted in accordance with the Declaration of Helsinki and approved by the Montreal Heart Institute's ethical committee (Montreal, QC, Canada; ethics No. ICM #01-406). All of the subjects provided written informed consent to the experimental protocol before participating in the study. Venous blood was obtained from healthy donors free from medication for at least 10 days prior to the experiments. Venous blood was obtained by drawing 100 ml (4×25 ml) of blood using a 21G needle into 30 ml syringes prefilled with 5 ml of Anticoagulant Citrate Dextrose Solution USP (ACD) Formula A (Baxter Healthcare; Deerfield, IL). The blood was then transferred into 4×50 ml tubes and spun for 15 min at 200 g at room temperature. Following the centrifugation, the platelet rich plasma (PRP) was removed from the top layer and 20 ml of a 4% Dextran solution (138 mM NaCl, 5 mM KCl, 0.34 mM Na_2_HPO_4_, 0.4 mM KH_2_PO_4_, 4.2 mM NaHCO_3_, 5.6 mM Glucose, 10 mM HEPES, 12.9 mM Sodium Citrate and 250 mM Dextran; pH 7.4) was added per tube. The tubes were gently mixed and red blood cells were left to sediment for 45 minutes at room temperature. The upper layer containing the white blood cells was collected and gently deposed on a 12.5 ml layer of Ficoll-Paque Plus (GE Healthcare; Baie d'Urfé, QC, Canada) in 50 ml tubes and spun for 28 minutes at 400 g and at room temperature [24]–[26]. Following this centrifugation, the monocytes and lymphocytes were separated from the neutrophils by Ficoll gradient. The reminiscent red blood cells and neutrophils were found in the pellet. In order to eliminate the red blood cells from the neutrophils, we used a water lysis procedure by which we added 20 ml of distilled water over the neutrophils and red blood cells pellet and mix gently for 20 seconds, followed by the quick addition of 20 ml of HBSS 2X solution while continuing mixing, for a final concentration of HBSS 1X (pH 7.4). Neutrophils were then spun for 10 minutes at 200 g and at room temperature. The pellet was then resuspended in RPMI 1640 medium with Corning Glutagro (Mediatech, Manassas, VA) supplemented with 25 mM HEPES (N-2-hydroxyethylpiperazine-N′-2-ethanesulfonic acid) and 1% penicillin/streptomycin. Contamination of isolated neutrophil suspension with peripheral blood mononuclear cells was less than 0.1% as determined by morphological analysis and flow cytometry, and viability was found to be greater than 98%, as assessed by Trypan blue dye exclusion assay.

### RNA studies

Two RT-qPCR -based techniques were used. The first of these is a gene-based screening method; more specifically, real time quantitative polymerase chain reaction (RT-qPCR) arrays were used to identify targets of angiopoietins stimulation in inflammation. The second method was used to confirm array results and to expand mRNA expression kinetics. Recombinant human Ang1 and Ang2 were obtained from R&D Systems (Minneapolis, MN) and bacterial lipopolysaccharide (LPS) from Sigma-Aldrich (St Louis, MO).

#### RT-qPCR array analyses

Neutrophils (10^7^ cells/ml; 1 ml) from at least three independent donors were treated with PBS, Ang1 (10^−8^ M) or Ang2 (10^−8^ M) for 90 minutes prior to DNAse treatment and total RNA extraction with the RNeasy extraction kit (Qiagen, Mississauga, ON, Canada). RNA samples were evaluated for integrity using a Bioanalyzer 2000 system (Genome Quebec Innovation Centre, McGill University, Montréal, QC, Canada); when all three samples (PBS, Ang1 and Ang2) from the same donor showed an mRNA integrity above 8.5, they were selected for use in arrays. RNA integrity between selected samples differed by less than 0.5. Following isolation, 2 µg of RNA were processed with RT^2^ First Strand Kit (SA Biosciences, Frederick, MD) according to manufacturer's instructions. Quantitative PCR analyses of chemokines and receptors were assessed with the Chemokines & Receptors PCR Array (SA Biosciences), RT^2^ SYBR® Green qPCR master mix (SA Biosciences) and a Stratagene Mx3500p qPCR System (Stratagene, La Jolla, CA). PCR array data were analyzed by the RT^2^ Profiler PCR Array Data Analysis program, available through SA Biosciences' web portal and based on the ΔΔCt method with four different housekeeping genes. Data were normalized to 4 housekeeping genes (B2M, HPRT1, RPL13A and GAPDH) and represented in a volcano plot of fold change in expression of each gene (compared to PBS-control) against its p-value.

#### RT-qPCR kinetics

Total RNAs (100 ng) from PBS, LPS (1 µg/ml), Ang1 (10^−9^ and 10^−8^ M) or Ang2 (10^−9^ and 10^−8^ M)-treated neutrophils were extracted as mentioned above and reverse transcribed using random hexamers and the Moloney murine leukemia virus (MMLV) reverse transcriptase (Invitrogen, Burlington, ON, Canada) according to manufacturer's instructions. Reactions were carried out on a MX3500P (Stratagene) using 10 ng of cDNAs, Syber Green (Invitrogen) and 300 nM of specific primers as follows (5′ to 3′): • IL-1α forward (Fwd) TGACCTGGAGGCCATCGCCAA; reverse (Rev) GCAGCAGCCGTGAGGTACTGA, • IL-1β Fwd ACGCTCCGGGACTCACAGCA; Rev TGAGGCCCAAGGCCACAGGT, • IL-1RA Fwd GATGTGGTACCCATTGAGCCTCATGC; Rev ACTGGTGGTGGGGCCACTGT. cDNAs were submitted to 40 cycles of amplification (temperatures for annealing: 60°C; dissociation: 55°C) and gave single peaks for each product. RT-qPCR products were purified on a 2% acrylamide gel, quantified using QIAquick Gel Extraction Kit (Qiagen) and sequenced. Gene expression was normalized using β-microglobulin as the housekeeping gene and results were expressed relative to calibrator T_0_ (gene expression at time of isolation) or to the control-PBS at each time point.

In another set of experiments, neutrophils were pretreated with inhibitors of p38 MAP kinase (SB203580; 10 µM), MEK1/2 (U0126; 20 µM), Akt (Triciribine; 5 µM), DMSO (vehicle) or PBS for 30 minutes prior to a 1-hour stimulation with PBS, LPS (1 µg/ml), Ang1 (10^−10^–10^−8^ M) or Ang2 (10^−10^–10^−8^ M). Total RNAs were then extracted and submitted to RT-qPCR analyses as aforementioned.

### Quantification of cytokines by ELISA

Purified neutrophils (10^7^ cells/ml; 1 ml) were incubated in RPMI and treated with PBS, LPS (1 µg/ml) Ang1 (10^−10^–10^−8^ M) or Ang2 (10^−10^–10^−8^ M) for up to 24 hours at 37°C and 5% CO_2_. Upon the incubation period, neutrophils were centrifuged at 900 g for 6 minutes and supernatants collected and stored at −80°C. The centrifuged cells were then lysed in ice-cold 1% Triton-RPMI solution containing a cocktail of protease inhibitors. The complete kinetics of synthesis and release of IL-1α, IL-1β and IL-1RA as well as those for pro-IL-1β were evaluated from cell-lysates and supernatants respectively, using Quantikine (pro-IL-1β; R&D Systems) or Duoset ELISA development kits (IL-1α, 1β, 1RA; R&D Systems) and in accordance with manufacturer's instructions.

In another set of experiments, neutrophils were pretreated with DMSO-soluble inhibitors of p38 MAPK (SB203580; 1 and 10 µM), MEK1/2 (U0126; 2 and 20 µM), Akt (Triciribine; 1 and 5 µM), DMSO or PBS for 30 minutes prior to a 2-hour stimulation with PBS, LPS, Ang1 or Ang2. Final DMSO concentration in reaction volumes did not exceed 0.2%. Upon agonist stimulation, supernatants and lysates were collected and the concentrations of cytokines assessed by ELISA.

### IL-1β maturation

IL-1β is synthesized in the cytoplasm as a 31-kDa precursor pro-protein (pro-IL-1β) that is cleaved to its mature 17-kDa form by IL-1β-converting enzyme (ICE; also known as caspase-1). Neutrophils (10^7^ cells/ml; 1 ml) treated with PBS, LPS (1 µg/ml), Ang1 (10^−10^–10^−8^ M) or Ang2 (10^−10^–10^−8^ M) for up to 6 hours were assessed for caspase-1 activity using the Caspase-1 Fluorometric Assay (R&D Systems). Upon each incubation period, neutrophils were centrifuged at 900 g for 6 minutes and the supernatants were gently discarded. Cells were then lysed with 250 µl of lysis buffer (provided by the manufacturer) and protein concentrations were determined with the BCA protein assay. The enzymatic reaction for caspase-1 activity was carried out in a 96-well flat bottom microplate using 50 µl (150 µg) of total protein and the caspase-1 fluorogenic substrate WEHD-AFC. The plates were incubated at 37°C for 2 hours and read on a fluorescent microplate reader (excitation 400 nm, emission 505 nm).

In previous studies, Perregaux *et al.* reported that the potassium ionophore, nigericin is capable of inducing efficient cleavage and release of newly synthesized IL-1β from LPS-treated macrophages [27], [28]. We tested this hypothesis in 4 sets of neutrophils (10^7^ cells/ml; 1 ml), each set treated with PBS, LPS (1 µg/ml), Ang1 (10^−8^ M) or Ang2 (10^−8^ M), for 2 hours at 37°C and 5% CO_2_ to induce maximal accumulation of pro-IL-1β. Upon the first incubation period, neutrophils were centrifuged at 900 g for 6 minutes. Lysates and supernatants were immediately collected from one set; for the remaining three sets, supernatants were carefully removed and replaced with RPMI containing vehicle (DMSO + ethanol), Cycloheximide (CHX; 10 µg/ml; + ethanol) to halt new protein synthesis, or CHX and the potassium-proton ionophore nigericin (N; 20 µM) for a further 45 minutes treatment (37°C and 5% CO_2_). Upon this second incubation period, the three sets were centrifuged at 900 g for 6 minutes, and supernatants and lysates were collected and assayed for pro-IL-1β and IL-1β concentrations by ELISA, as previously described. CHX was dissolved in DMSO to a final DMSO concentration that did not exceed 0.1%. Nigericin was dissolved in ethanol to a final ethanol concentration that did not exceed 0.05%.

### Statistical analyses

Results are presented as the mean ± SEM of independent experiments performed on neutrophils of at least three independent donors. Statistical comparisons were made by one-way analysis of variance (ANOVA) followed by a Dunnett or Tukey post-hoc test where applicable using GraphPad Prism (Mac version 5.0b). Differences were considered significant at p values less than 0.05.

## Results

### Expression of inflammatory cytokines and their receptors

**Figure 1:**
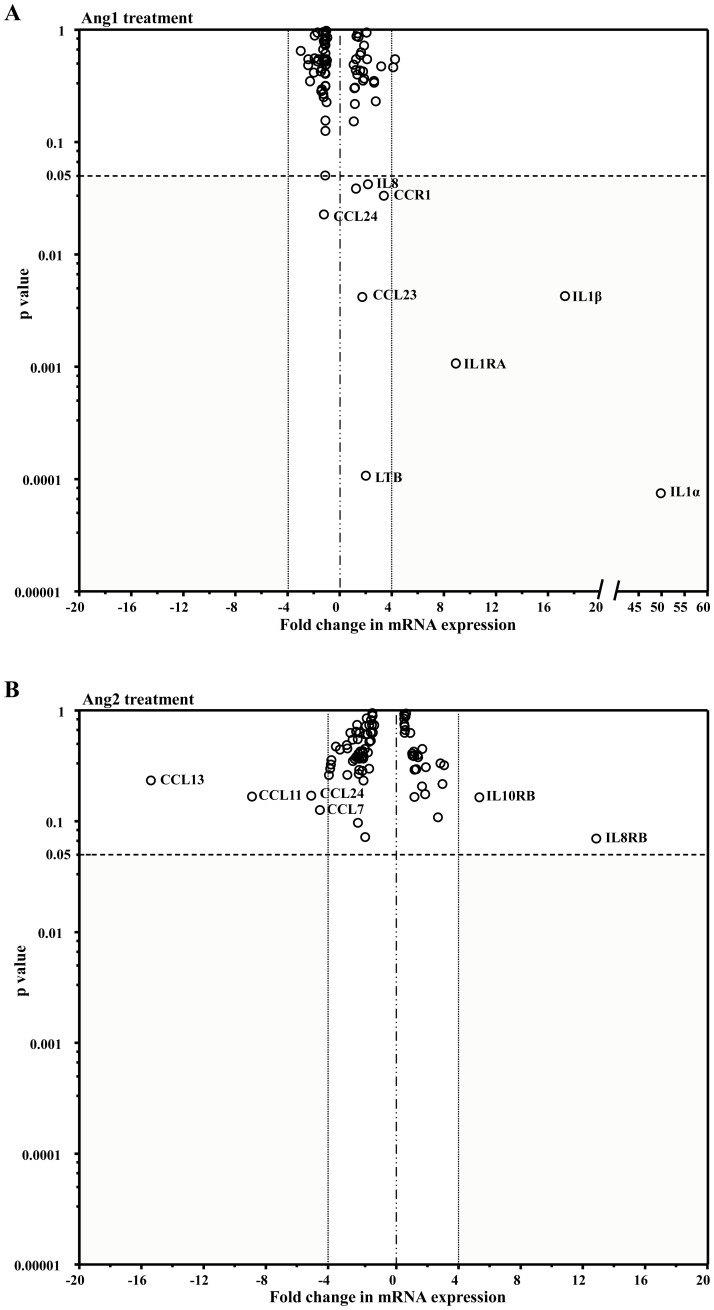
Expression of inflammatory cytokines and their receptors in neutrophils. Circulating neutrophils isolated from 6 different donors were treated with PBS, Ang1 (10^−8^ M) (**A**) or Ang2 (10^−8^ M) (**B**) for 90 minutes prior to RNA isolation. Data are expressed in a Volcano plot, as fold change in gene expression (x-axis) compared to PBS-treated cells; values outside the dotted vertical lines indicate significant fold increases (positive values) or fold decreases (negative values). Values below the dashed horizontal line (p<0.05) underline statistical significance (y-axis). Each circle corresponds to the fold-expression of a single gene.

We have recently reported that Ang1 (10^−8^ M) promotes the synthesis and release of the inflammatory cytokine IL-8 from neutrophils within 60 minutes and peaking within 2 hours of stimulation, whereas Ang2 has no such effect [12]. Extending these observations, we assessed the potential of angiopoietins to modulate the expression of 84 inflammatory cytokines in neutrophils. Neutrophils were treated with PBS (control vehicle), Ang1 or Ang2 (10^−8^ M; 90 minutes), and total mRNAs were extracted for RT-qPCR array analyses. For follow-up experiments, we selected genes with a nominal p-value <0.05 and a change in expression level ≥ 4-fold (Figure 1A and B).

Based on the above criteria, most genes that were assayed did not fluctuate significantly following treatment with either angiopoietins (see Tables 1 and 2 for a list of all genes and their p-value). However, Ang1, but not Ang2, significantly upregulated the expression of 3 genes belonging to the IL-1 family (Figure 1A): IL-1α (49.65-fold increase; p<0.001), IL-1β (17.23-fold increase; p<0.01) and the endogenous antagonist IL-1RA (8.85-fold increase; p<0.01) as compared to PBS-treated cells. Neither other members of the IL-1 family nor the biologically active receptor IL-1R1 varied significantly under our experimental conditions (Table 1).

**Table 1:** Expression change of inflammatory cytokines and their receptors following Ang1 treatment.

Genes	Fold	P	Genes	Fold	P	Genes	Fold	P	Genes	Fold	P
ABCF1	2.58	0.36	CCL4	1.12	0.31	CXCL14	−1.16	0.16	IL1F9	−1.18	0.05
BCL6	1.83	0.37	CCL5	−1.29	0.26	CXCL2	1.51	0.60	IL-1R1	2.04	0.56
C3	−1.26	0.56	CCL7	−1.78	0.53	CXCL3	1.51	0.44	IL-1RA	8.85	0.001
C4A	−1.04	0.54	CCL8	−1.16	0.13	CXCL5	−1.22	0.77	IL22	−1.32	0.68
C5	−1.16	0.32	CCR1	3.33	0.04	CXCL6	1.73	0.43	IL5	−1.16	0.75
CCL1	−1.16	0.41	CCR2	−1.47	0.29	CXCL9	−1.16	0.90	IL5RA	−1.34	0.97
CCL11	−3.05	0.66	CCR3	4.19	0.56	CARD18	−1.16	0.32	IL8	2.11	0.04
CCL13	−2.47	0.56	CCR4	−1.16	0.54	IFNA2	1.11	0.22	IL8RA	1.23	0.89
CCL15	−1.16	0.56	CCR5	1.02	0.16	IL10	−1.30	0.56	IL8RB	2.72	0.23
CCL16	−1.16	0.84	CCR6	−1.28	0.81	IL10RA	1.19	0.56	IL9	−1.30	0.96
CCL17	−1.09	0.50	CCR7	1.01	0.50	IL10RB	4.05	0.56	IL9R	−1.19	0.42
CCL18	−1.16	0.82	CCR8	−1.43	0.30	IL13	−2.05	0.42	LTA	−1.42	0.46
CCL19	1.73	0.36	CCR9	−1.16	0.49	IL13RA1	1.29	0.40	LTB	1.95	0.0001
CCL2	2.59	0.34	CEBPB	1.07	0.31	IL17C	−2.33	0.35	LTB4R	1.17	0.44
CCL20	3.13	0.48	CRP	−1.32	0.27	IL-1α	49.65	0.000008	MIF	−1.07	0.23
CCL21	−2.48	0.49	CX3CR1	1.33	0.94	IL-1β	17.24	0.004	SCYE1	−1.01	0.87
CCL23	1.67	0.00	CXCL1	2.01	0.96	IL1F10	−1.16	0.96	SPP1	1.39	0.88
CCL24	−1.27	0.02	CXCL10	−1.09	0.99	IL1F5	−1.14	0.96	TNF	1.19	0.04
CCL25	−1.17	0.56	CXCL11	−1.16	0.53	IL1F6	−1.16	0.96	CD40LG	−1.16	0.63
CCL26	−1.99	0.57	CXCL12	−1.98	0.90	IL1F7	1.58	0.64	TOLLIP	1.82	0.74
CCL3	−1.10	0.56	CXCL13	−1.67	0.56	IL1F8	−1.76	0.96	XCR1	−1.50	0.43
B2M*	1.24	0.56	HPRT1*	1.07	0.47	RPL13A*	−1.34	0.40	GAPDH*	1.01	0.04

Human neutrophils from at least 3 different individuals were treated with PBS, Ang1 or Ang2 (Table 2) at 10^−8^ M for 90 minutes. RT-qPCR array analyses were performed to assess expression change of 84 genes involved in the inflammatory response. Each gene from angiopoietin-treated neutrophils was compared to PBS-treated neutrophils and the data expressed as fold change. Negative and positive values denote a decrease and increase in mRNA expression, respectively. Differences were considered significant at Fold ≥ 4 and p<0.05. Housekeeping genes are denoted by an asterisk (*). Members of the IL-1 family that satisfied both requirements were considered significantly upregulated by Ang1, and are shaded in grey.

**Table 2:** Expression change of inflammatory cytokines and their receptors following Ang2 treatment.

Genes	Fold	P	Genes	Fold	P	Genes	Fold	P	Genes	Fold	P
ABCF1	1.48	0.39	CCL4	2.19	0.21	CXCL14	−1.63	0.44	IL1F9	−1.45	0.73
BCL6	1.23	0.64	CCL5	1.04	0.76	CXCL2	3.39	0.34	IL-1R1	1.10	0.68
C3	1.73	0.39	CCL7	−4.43	0.13	CXCL3	1.09	0.91	IL-1RA	1.16	0.95
C4A	−1.09	0.75	CCL8	−1.34	0.62	CXCL5	−3.15	0.34	IL22	−3.17	0.27
C5	−1.16	0.64	CCR1	−1.13	0.64	CXCL6	1.71	0.29	IL5	−1.54	0.40
CCL1	−1.56	0.45	CCR2	1.65	0.43	CXCL9	1.51	0.45	IL5RA	−2.30	0.55
CCL11	−8.00	0.17	CCR3	3.52	0.22	CARD18	−1.47	0.62	IL8	3.21	0.11
CCL13	−12.29	0.24	CCR4	−1.02	0.91	IFNA2	1.06	0.64	IL8RA	2.21	0.46
CCL15	−1.56	0.45	CCR5	−1.31	0.42	IL10	−2.64	0.26	IL8RB	13.48	0.07
CCL16	−1.68	0.45	CCR6	−1.05	0.75	IL10RA	1.12	0.90	IL9	−1.68	0.29
CCL17	−1.24	0.30	CCR7	2.39	0.18	IL10RB	5.03	0.17	IL9R	−1.96	0.12
CCL18	−1.79	0.44	CCR8	−1.80	0.43	IL13	−3.76	0.30	LTA	−1.90	0.27
CCL19	−1.40	0.86	CCR9	−1.22	0.53	IL13RA1	1.70	0.17	LTB	−1.74	0.39
CCL2	−1.99	0.38	CEBPB	−1.51	0.45	IL17C	−2.46	0.64	LTB4R	1.82	0.30
CCL20	1.91	0.40	CRP	−1.91	0.67	IL-1α	3.63	0.32	MIF	−2.01	0.75
CCL21	−2.29	0.35	CX3CR1	−1.23	0.75	IL-1β	2.44	0.31	SCYE1	1.07	0.73
CCL23	1.03	0.94	CXCL1	−1.84	0.64	IL1F10	−1.83	0.44	SPP1	1.55	0.41
CCL24	−4.98	0.17	CXCL10	−1.03	0.96	IL1F5	−1.61	0.23	TNF	−2.69	0.49
CCL25	−1.12	0.83	CXCL11	−1.51	0.44	IL1F6	−1.83	0.44	CD40LG	−1.02	0.60
CCL26	−3.40	0.48	CXCL12	−3.68	0.36	IL1F7	−1.97	0.56	TOLLIP	1.57	0.39
CCL3	1.03	0.86	CXCL13	−3.13	0.45	IL1F8	−1.89	0.29	XCR1	−2.64	0.46
B2M*	1.05	0.74	HPRT1*	−1.22	0.94	RPL13A*	−1.34	0.93	GAPDH*	1.56	0.21

As per Table 1, each gene from Ang2-treated neutrophils was compared to its counterpart from PBS-treated neutrophils and the data expressed as fold-change. Although genes such as CCL7, CCL11, CCL13, CCL24 and IL10RB showed substantial fold-change differences between Ang2 and PBS, statistical significance denoted by the p-value was far from 0.05. Because of its fold-regulation and a p-value close to 0.05, IL-8RB is a promising target. Housekeeping genes are denoted by an asterisk (*).

Under less stringent statistical parameters (p≈0.05 and gene modulation ≥ 2-fold change), qPCR arrays identified three potentially interesting targets of Ang1 treatment: IL-8/CXCL8, Lymphotoxin Beta (LTB) and C-C chemokine receptor type 1 (CCR1) (Table 1 and Figure 1A). In parallel, Ang2 showed a tendency to up-regulate IL-8 receptor B (IL-8RB)/CXCR2 (Table 2 and Figure 1B). The significance of these potential targets will be covered briefly in the discussion.

### Effect of angiopoietins on the mRNA expression of IL-1α, IL-1β and IL-1RA

Given the strong response of neutrophils in up-regulating IL-1 expression, and the importance of the latter family in initiating and modulating the inflammatory response, we sought to confirm and expand on the above using custom primers for IL-1α, IL-1β and IL-RA. Kinetics were performed by treating neutrophils with PBS, Ang1 (10^−10^–10^−8^ M), Ang2 (10^−10^–10^−8^ M) or LPS (1 µg/ml; positive control), for up to 6 hours before mRNA extraction. Given that lower concentrations of angiopoietins (10^−10^–10^−9^ M) had no significant effect on mRNA expression compared to PBS-control, only the highest concentration of the angiopoietins (10^−8^ M) is represented in the graphs throughout the study. Additionally, because maximal Ang2 (10^−8^ M) had no significant effect compared to PBS-control, only Ang1 (10^−8^ M) is discussed below.

**Figure 2:**
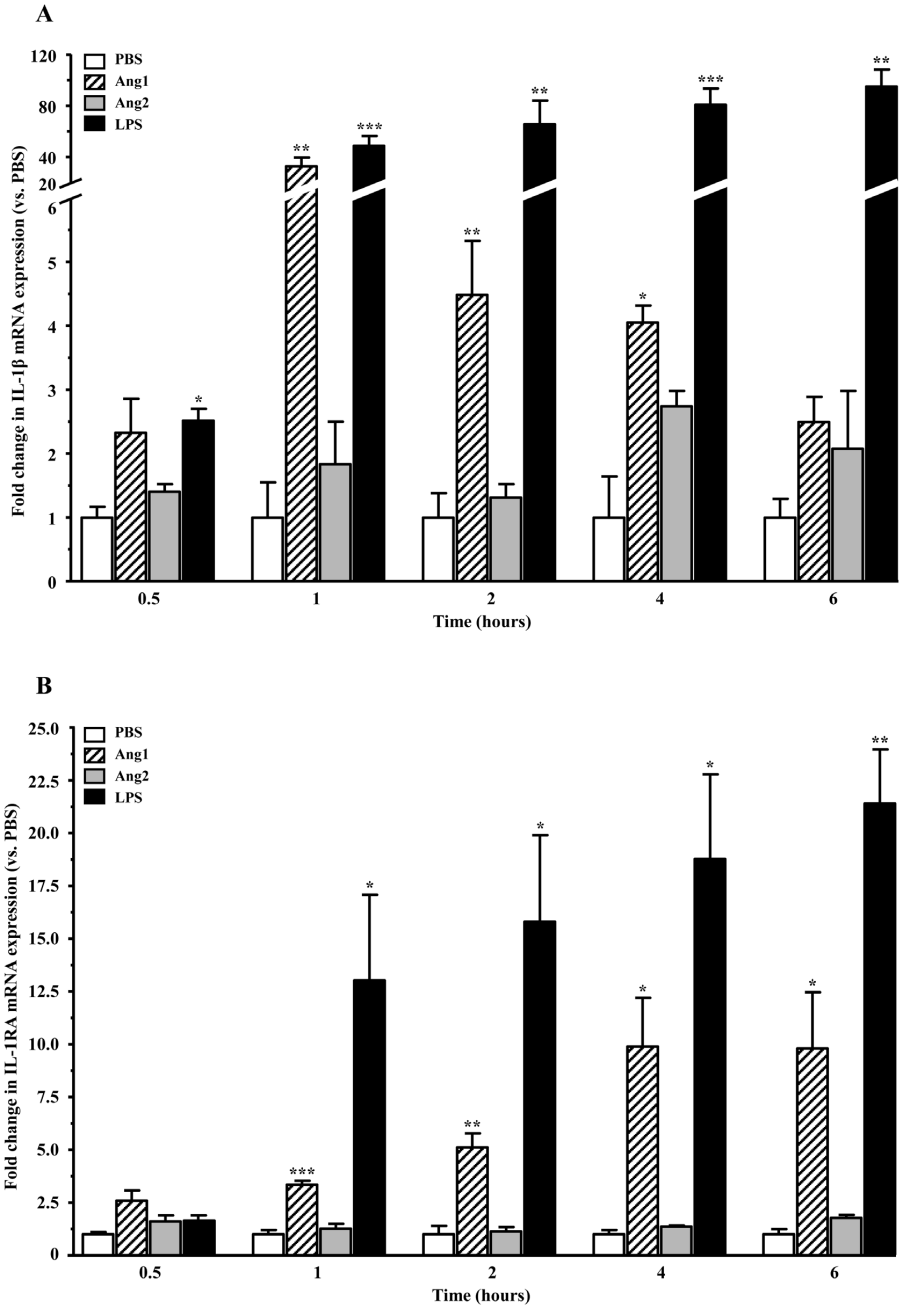
Kinetics of mRNA expression of IL-1β and IL-1RA. Primers were designed to quantify changes in the mRNA levels of IL-1β (**A**) and IL-1RA (**B**), following treatment with PBS, angiopoietins (10^−8^ M) or LPS (1 µg/ml), for up to 6 hours. For each time point, basal (PBS) mRNA expression is set to unitary value, and the data are presented as fold change compared to its corresponding PBS. For angiopoietins, only values resulting from treatment with the highest concentration (10^−8^ M) are shown. Data are represented as the means ± SEM of at least three independent experiments. *p<0.05, **p<0.01, ***p<0.001 vs. PBS-control (Dunnett's test).

IL-1β mRNA was abundantly expressed in neutrophils, with a cycle threshold (Ct)<25 at the time of isolation (T_0_), and basal (PBS) IL-1β mRNA did not significantly change over time. Treatment with Ang1 (10^−8^ M) induced a rapid increase in IL-1β mRNA expression within an hour of stimulation, with 32.6-fold increase compared to PBS-treated cells, after which expression progressively returned to basal values (Figure 2A). Levels of IL-1RA mRNA were also abundant in neutrophils, with a Ct<25 at T_0_. Similarly to the potent positive control LPS, Ang1 promoted a significant increase in IL-1RA mRNA expression as early as 1 hour after stimulation, with 3.3-fold expression increase over PBS-control and reaching up to 9.8-fold at 6 hours (Figure 2B). Finally, regardless of treatment, IL-1α mRNA levels were hardly detectable in neutrophils (Table 3), with a Ct>42 at T_0_. Subsequent basal and treated Ct values remained above 35 throughout the 6-hour time-period indicating that IL-1α mRNA is barely, if at all, expressed in neutrophils.

**Table 3:** IL-1 mRNA and protein expression in freshly isolated and Ang1-stimulated neutrophils.

Genes	mRNA	Protein		
		T_0_ Intracellular (S) Extracellular (S)		
IL-1α	N.D.	N.D	N.D	N.D
IL-1β	+	N.D	+	N.D
Pro-IL-1β	+	N.D	+	N.D
IL-1RA	+	+	+	+

T_0_ represents content at the time of isolation and reflects the state of circulating human neutrophils in healthy individuals; intracellular and extracellular content are assessed after stimulation with Angiopoietin-1 (Ang1); S: stimulated; N.D.: not detectable. Unstimulated neutrophils do not express or store pools of IL-1α mRNA, and that is not altered by the addition of Ang1. Neutrophils hold large pools of IL-1β mRNA, but Ang1 signal is required for translation. Finally, neutrophils constitutively express and release IL-1RA.

### Kinetics of protein synthesis and release

Building on the mRNA kinetics studies, we assessed basal expression and *de novo* protein synthesis and release for all three IL-1 family members following angiopoietin treatment. Kinetics studies were extended to a 24-hour period; at each time point, using the same lysates and/or supernatants, the concentrations of IL-1α, IL-1β or IL-1RA protein were simultaneously evaluated by ELISA. For the same reasons as per the mRNA section, only the highest concentration of Ang1 (10^−8^ M) is discussed below.

**Figure 3:**
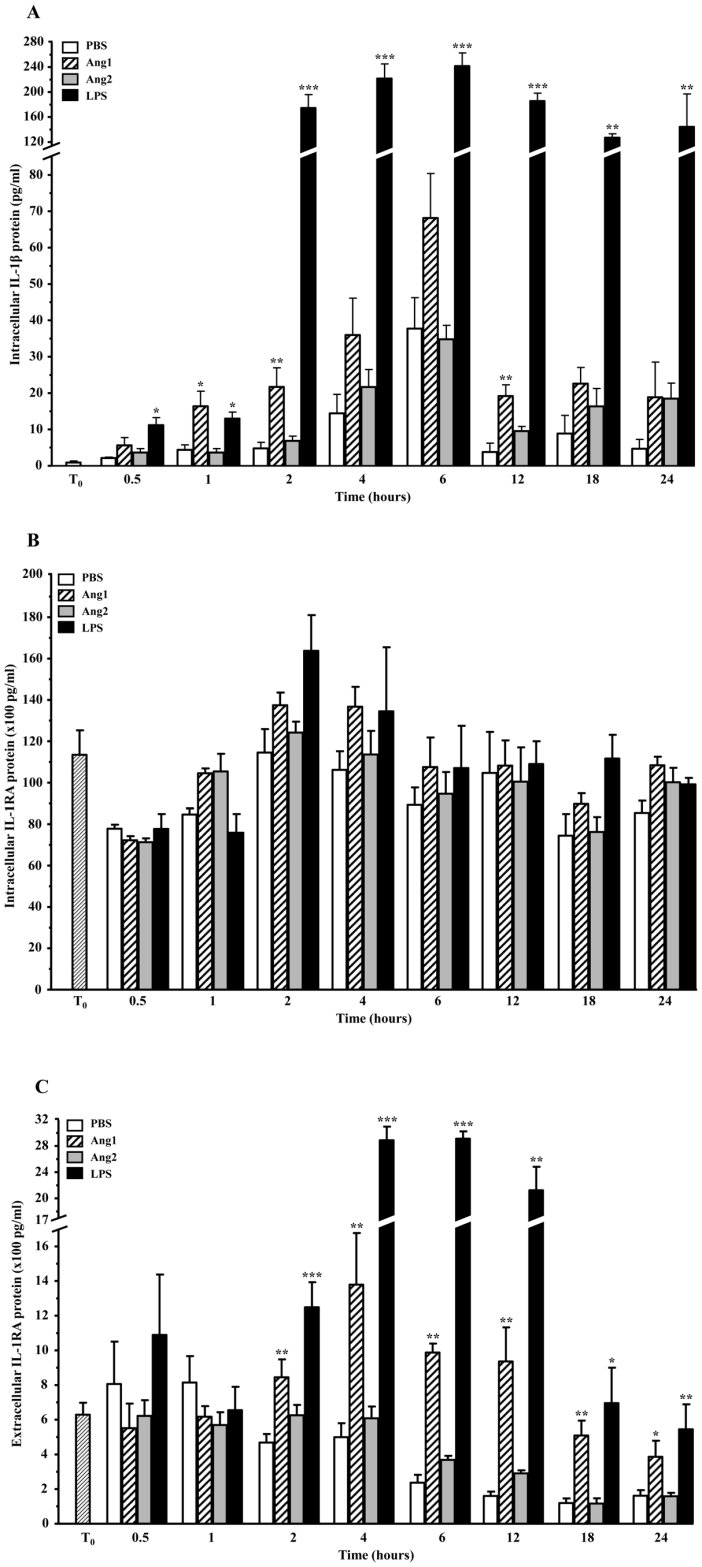
Kinetics of IL-1β and IL-1RA protein expression. Neutrophils were treated with PBS, angiopoietins (10^−8^ M) or LPS (1 µg/ml), for up to 24 hours. Intracellular IL-1β (**A**), IL-1RA (**B**) and extracellular IL-1RA (**C**) were quantified by ELISA. For angiopoietins, only values resulting from treatment with the highest concentration (10^−8^ M) are shown. No IL-1β was detected extracellularly. Data are represented as the means ± SEM of at least three independent experiments. *p<0.05, **p<0.01, *** p<0.001 vs. PBS-control (Dunnett's test).

Intracellular levels of IL-1β in neutrophils (10^7^ cells/ml) were almost undetectable at T_0_ (Figure 3A). Basal IL-1β protein levels in PBS-treated neutrophils fluctuated over time, starting with 2.1 pg/ml at 30 minutes, reaching a peak of 37.7 pg/ml at 6 hours and declining to 4.7 pg/ml at 24 hours. Ang1 (10^−8^ M) treatment lead to a steady increase in IL-1β synthesis throughout the first 6 hours of stimulation, going from 16.4 pg/ml at 1 hour, up to 68.1 pg/ml at 6 hours, and then stabilizing between 12 and 24 hours at a value below 22 pg/ml.

Several studies performed on macrophages and monocytes *in vitro* reported that LPS and other mediators are capable of promoting IL-1β protein synthesis but fail to induce IL-1β release [27]–[31]. Our current study shows that circulating human neutrophils behave in much of the same manner; indeed, under all conditions tested, no IL-1β was detected in the supernatants (Table 3), suggesting that the decreases in intracellular IL-1β levels over time were not due to its release.

Unlike IL-1β, intracellular levels of IL-1RA were substantial at T_0_, with detection at 11 350 pg/ml (Figure 3B). We observed a short drop in intracellular IL-1RA in the first 30 minutes of stimulation, with levels fluctuating between 7 130–7 800 pg/ml regardless of treatment. For the remainder of the time-course, variations in intracellular IL-1RA levels were not statistically significant between treatments (Figure 3B), averaging between 7500 pg/ml to up to 16 400 pg/ml. In another stark contrast to IL-1β, we observed that neutrophils constitutively release IL-1RA: At T_0_, we detected 629.1 pg/ml of extracellular IL-1RA in the supernatants (Figure 3C). Detection of extracellular IL-1RA under basal conditions continued throughout the entire time-course and corresponded to about 2–10% of total IL-1RA cellular content. Similarly to LPS, Ang1 promoted a statistically significant increase in IL-1RA release as early as 2 hours following stimulation, with 844.6 pg/ml IL-1RA released (vs. 468.2 pg/ml for PBS), after which detection values climbed to a peak of 1 379.3 pg/ml at 4 hours (vs. 498.8 pg/ml for PBS) (Figure 3C). Finally, IL-1α protein was not detected in neutrophil cell lysates or in their corresponding supernatants at T_0_ or throughout the time-course under basal conditions or angiopoietin stimulation (Table 3).

### Is IL-1β a product of de novo synthesis or maturation from pre-existing pools of precursor?

IL-1β is synthesized in the cytoplasm as an inactive 31-kDa-precursor protein (pro-IL-1β) before being cleaved to its mature 17-kDa form [32]. Thus, we looked at the modulation of precursor pro-IL-1β levels in human neutrophils, and performed an initial assessment of the possible mechanisms governing pro-IL-1β cleavage. For the same reasons as per the mature protein, only the highest concentration of Ang1 (10^−8^ M) is discussed below.

#### De novo synthesis

**Figure 4:**
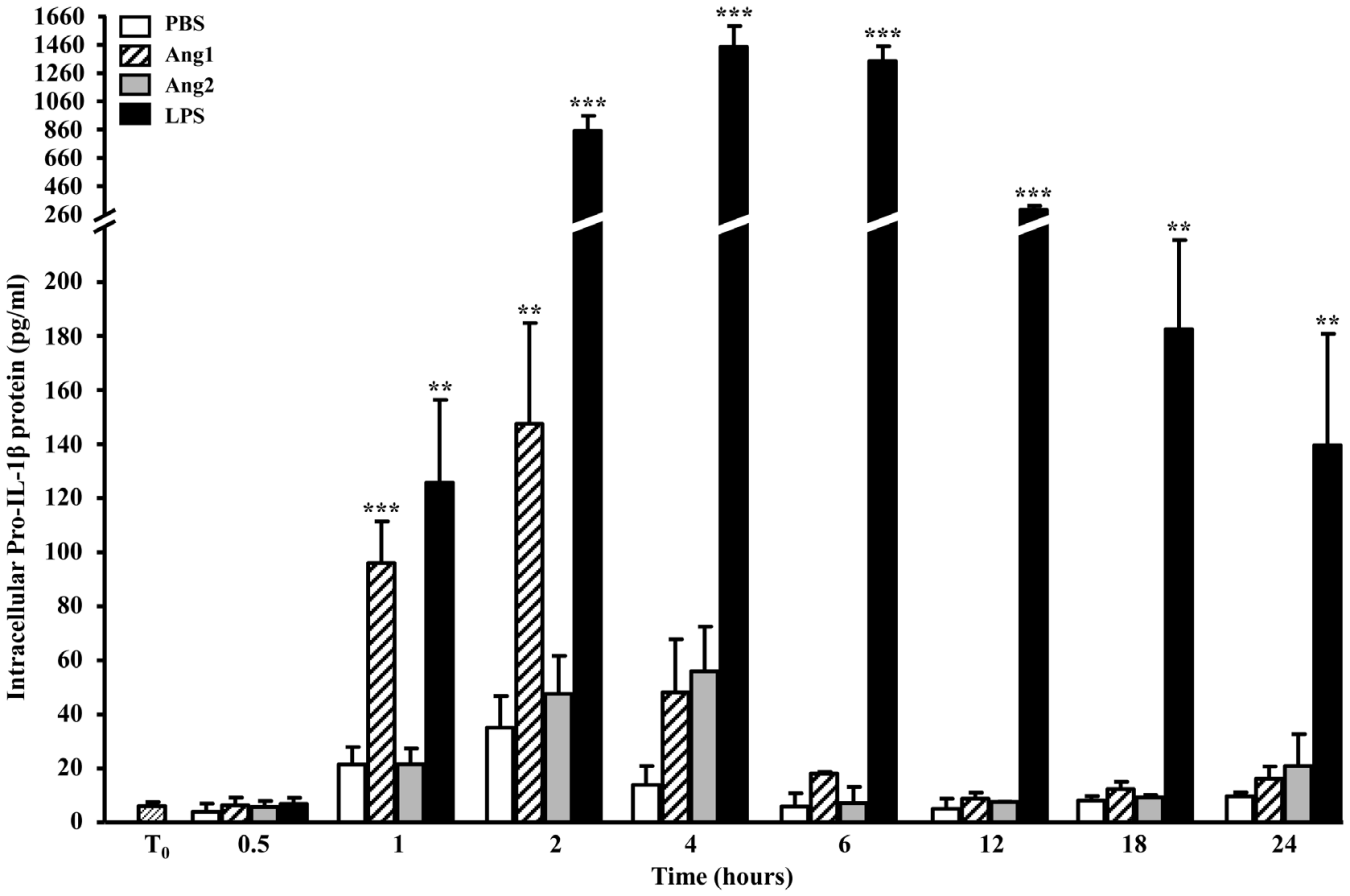
Kinetics of pro-IL-1β protein expression. Neutrophils were treated with PBS, angiopoietins (10^−8^ M) or LPS (1 µg/ml), for up to 24 hours. Only intracellular levels of pro-IL-1β were detectable, as no pro-IL-1β was detected in the supernatants at any time points and under any of the conditions tested. For angiopoietins, only values resulting from treatment with the highest concentration (10^−8^ M) are shown. Data are represented as the means ± SEM of at least three independent experiments. *p<0.05, **p<0.01, *** p<0.001 vs. PBS-control (Dunnett's test).

As per the mature protein, intracellular levels of pro-IL-1β in human neutrophils were almost undetectable at T_0_ (Figure 4). We observed an increase in pro-IL-1β levels under basal (PBS) conditions, reaching as much as 35.1 pg/ml at 2 hours, but subsequently declining to less than 10.0 pg/ml at 24 hours. Treatment with Ang1 (10^−8^ M) promoted a substantial increase in pro-IL-1β synthesis starting at 1 hour, with detection reaching a peak of 147.6 pg/ml at 2 hours, and then declining to 16.0 pg/ml at 24 hours, compared to PBS. Irrespective of the treatment, we did not detect pro-IL-1β proteins in the supernatant of neutrophils (Table 3), consistent with reports that pro-IL-1β is not released from cells [29], [30], [32]-[34].

Maturation of pro-IL-1β has been attributed primarily to the activation of caspase-1; however, independent studies reported that the processing of IL-1β might actually occur via a caspase-1-independent mechanism, through enzymes such as serine proteases Cathepsin G, Neutrophil Elastase and Proteinase-3 [35]–[38]. Using a fluorometric method, we assessed the activity of caspase-1 in human neutrophils treated with PBS, angiopoietins and LPS (1 µg/ml) for up to 6 hours. We did not detect any basal caspase-1 activity beyond threshold, and little to no changes following agonist stimulation (data not shown).

### Induction of pro-IL-1β maturation and IL-1β release

Studies have reported that the mechanisms leading to the maturation and effective release of IL-1β depend on the subset of leukocytes being investigated. While monocytes readily release IL-1β under LPS treatment [39], [40], macrophages require a depletion of intracellular potassium induced by ionophores such as nigericin before efficient IL-1β maturation and subsequent release [27], [28]. Because the mechanisms governing IL-1β maturation and release have never been reported in neutrophils, and given that even LPS failed at promoting IL-1β release, we tested the requirement for a secondary stimulus to drive neutrophil processing of pro-IL-1β and release of the mature protein.

**Figure 5:**
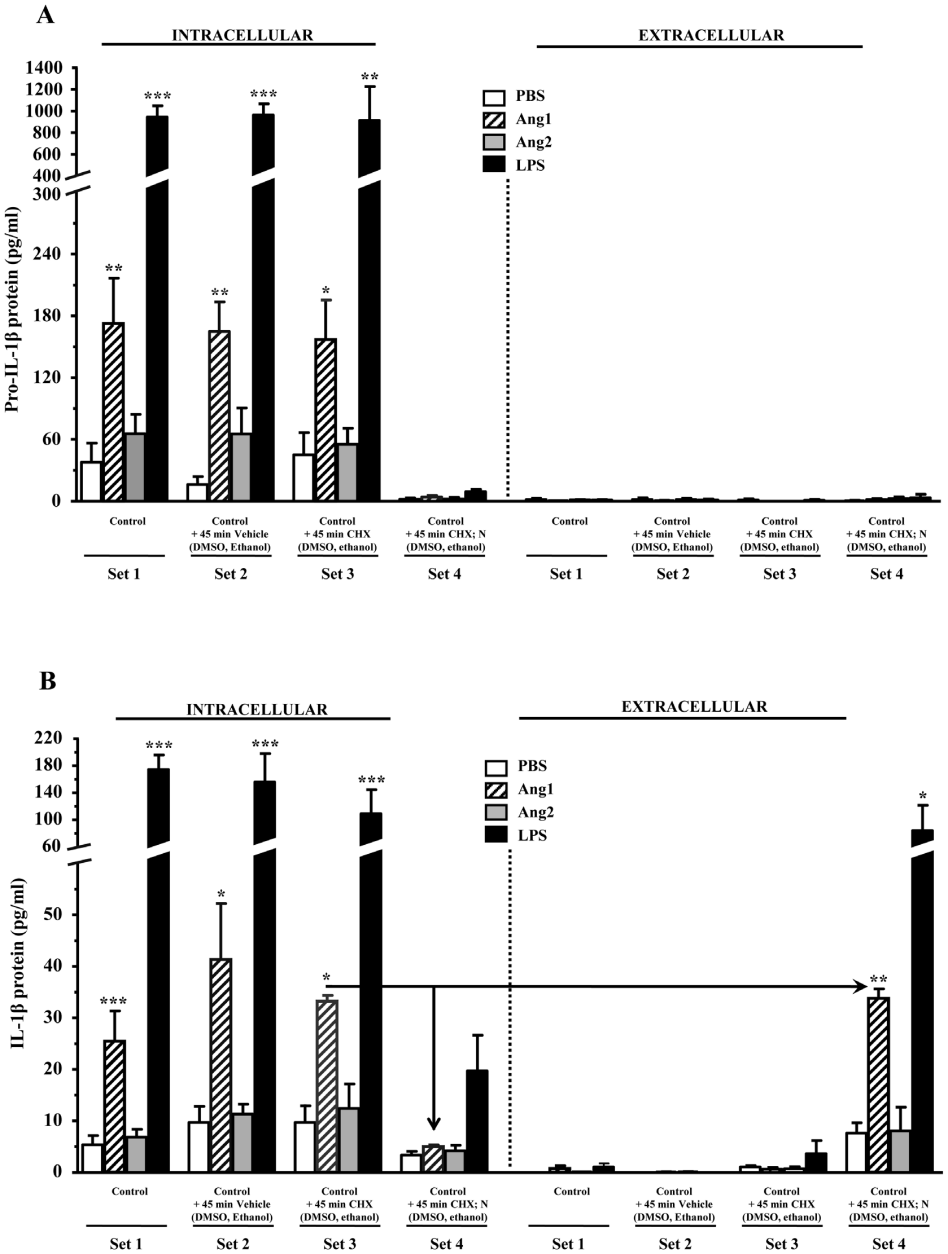
Effect of potassium depletion on IL-1β release. Neutrophils were treated with PBS, angiopoietins (10^−8^ M) or LPS (1 µg/ml), for two hours, followed by an additional 45-minute treatment with potassium ionophore nigericin, CHX, or appropriate vehicles. Changes in intracellular (left panels) and extracellular (right panels) levels of pro-IL-1β (**A**) and IL-1β (**B**) before and after ionophore addition were quantified by ELISA. CHX: Cycloheximide. N: Nigericin. Vehicles: DMSO, ethanol. Data are represented as the means ± SEM of at least three independent experiments. *p<0.05, **p<0.01, *** p<0.001 vs. PBS-control (Dunnett's test).

Neutrophils were divided into four sets (Figure 5; Sets 1-4) and were treated with agonists for 2 hours, a time when most of the new pro-IL-1β has already accumulated under Ang1 (refer to Figure 4). Upon this first incubation period, supernatants and cell lysates from Set 1 were collected. For Sets 2-4, supernatants were replaced with new media containing vehicles (DMSO + ethanol) or nigericin (N; in ethanol) for an additional 45 minutes as described in **Materials and Methods**. In order to eliminate the contribution of *de novo* synthesis to possible changes in levels of mature IL-1β (i.e. to confirm that any new IL-1β is a result of processing of the accumulated pro-IL-1β), a protein translation inhibitor, cycloheximide (CHX; in DMSO) was added during this step. To preserve sample comparability, supernatants from Sets 2-4 were supplemented with either DMSO or ethanol, as required.

Data from Sets 1 (Figure 5A and B) were used to establish the baseline of protein kinetics, and were comparable to what we had observed in our previous experiments for all the conditions tested. For pro-IL-1β (Figure 5A), neither the addition of vehicles (Set 2, left panel) nor CHX alone (Set 3, left panel) affected the synthesis of pro-IL-1β. Upon addition of nigericin (Set 4, left panel), we observed a near complete loss of detection of intracellular pro-IL-1β, in comparison to Set 3. This loss was not due to the release of pro-IL-1β, since the pro-protein in its native form was not detected in any of the corresponding extracellular fractions (Figure 5A, right panel).

Based on these observations, we hypothesized that nigericin may have indeed induced the processing of pro-IL-1β into IL-1β, as was reported to happen in macrophages [27], [28]. However, the concomitant evaluation of IL-1β levels indicated that this was not the case (Figure 5B): While the addition of nigericin (Set 4, left panel) almost completely depleted intracellular IL-1β content compared to neutrophils from Set 3, most of the IL-1β was recovered in the extracellular fraction of nigericin-treated neutrophils (Set 4, right panel). In fact, the amount of IL-1β recovered extracellularly from nigericin-treated neutrophils (Set 4, right panel) nearly matched what had accumulated inside the cells prior to nigericin treatment (Set 3, left panel). Thus, potassium depletion did not promote maturation of pro-IL-1β into IL-1β in human neutrophils, but only the selective exteriorization of IL-1β.

### Intracellular mechanisms of IL-1 family synthesis and release

Previous studies reported that the biological activities of angiopoietins can be mediated by PI-3K/Akt, p38 MAPK, and p42/44 MAPK pathways as a function of the cellular activities being solicited [11], [14], [41]–[43]. Thus, we wanted to delineate the signaling pathway(s) involved in mediating the effects of the angiopoietins on synthesis and/or secretion of IL-1 family members in human neutrophils. Neutrophils were pretreated with inhibitors of p42/44 MAPK Kinase - MEK1/2 - (U0126; (U0)), p38 MAPK (SB203580; (SB)) or Akt (Triciribine; (T)) for 30 minutes prior to agonist challenge, as previously described [12], [44]–[46]. Inhibitor-pretreated neutrophils were then compared to their vehicle (DMSO; D) counterparts.

#### mRNA changes

Because most of the inducible IL-1 mRNA was synthesized within the first hour of Ang1 treatment, we looked at the effects of the inhibitors on mRNA expression after 1 hour of agonist stimulation. Ang2 (10^−10^–10^−8^ M) and lower concentrations of Ang1 (10^−10^–10^−9^ M) yielded similar results as PBS-control under all conditions tested; thus, only the highest concentration of Ang1 (10^−8^ M) is discussed below.

**Figure 6:**
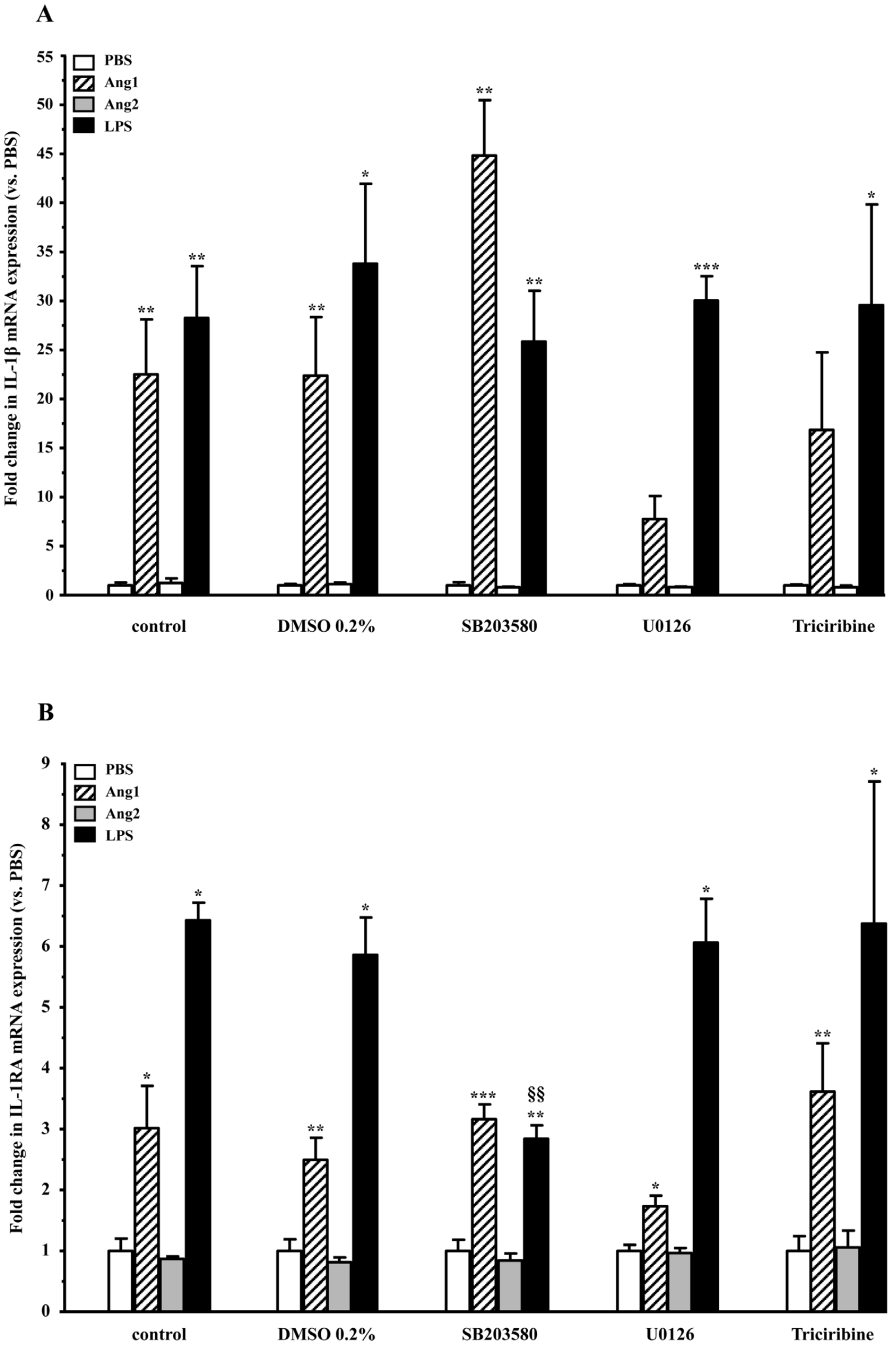
Effect of downstream signaling inhibitors on IL-1β and IL-1RA mRNA expression. Neutrophils were pretreated with inhibitors of Akt (Triciribine; 5 µM), p38 MAPK (SB203580; 10 µM), and p42/44 MAPKK (U0126; 20 µM), vehicle-DMSO (0.2%) or PBS for 30 minutes prior to a 1-hour agonist challenge. Total mRNA was used in RT-qPCR for assessment of mRNA expression of IL-1β (**A**) and IL-1RA (**B**). Data are presented as mean ± SEM of at least three independent experiments. *p<0.05, **p<0.01, *** p<0.001 vs. PBS-control within each set (Dunnett's test); §p<0.05, §§p<0.01 vs corresponding agonist-DMSO (Tukey's test).

Basal levels of IL-1β mRNA were not affected by either pretreatment with DMSO (vehicle) or with inhibitors (data not shown). Addition of the p38 MAPK inhibitor (SB) significantly increased the effect of Ang1 (10^−8^ M) on IL-1β mRNA expression, from 22.4- (Ang1-D) to 44.8-fold (Ang1-SB) expression (Figure 6A). MEK1/2 inhibition (U0) had the opposing effect, leading to a decrease from 22.4- to 7.8-fold (Ang1-U0) expression, corresponding to a 68% inhibition of Ang1 activity. The Akt inhibitor (T) had no significant effect on the activities of Ang1. Interestingly, none of the inhibitors significantly impacted the effects of LPS on IL-1β mRNA expression (Figure 6A).

As for IL-1RA mRNA expression, only MEK1/2 blockade provided a trend (not significantly) to decrease Ang1 effect (Figure 6B). On the other hand, the blockade of p38 MAPK activity significantly reduced the effect of LPS, from 5.9- (LPS-D) to 2.8-fold (LPS-SB) expression, corresponding to a 63% inhibition. The blockade of MEK1/2 or Akt pathways had no significant effects on the activities of LPS (Figure 6B).

#### Protein changes

The immediate impact of the aforementioned mRNA changes on the corresponding protein levels was assessed at 2 hours of agonist stimulation, coinciding with the time at which protein synthesis rate was also at its maximum. For the same reasons as per the mRNA experiments, only the highest concentration of Ang1 (10^−8^ M) is discussed below.

**Figure 7:**
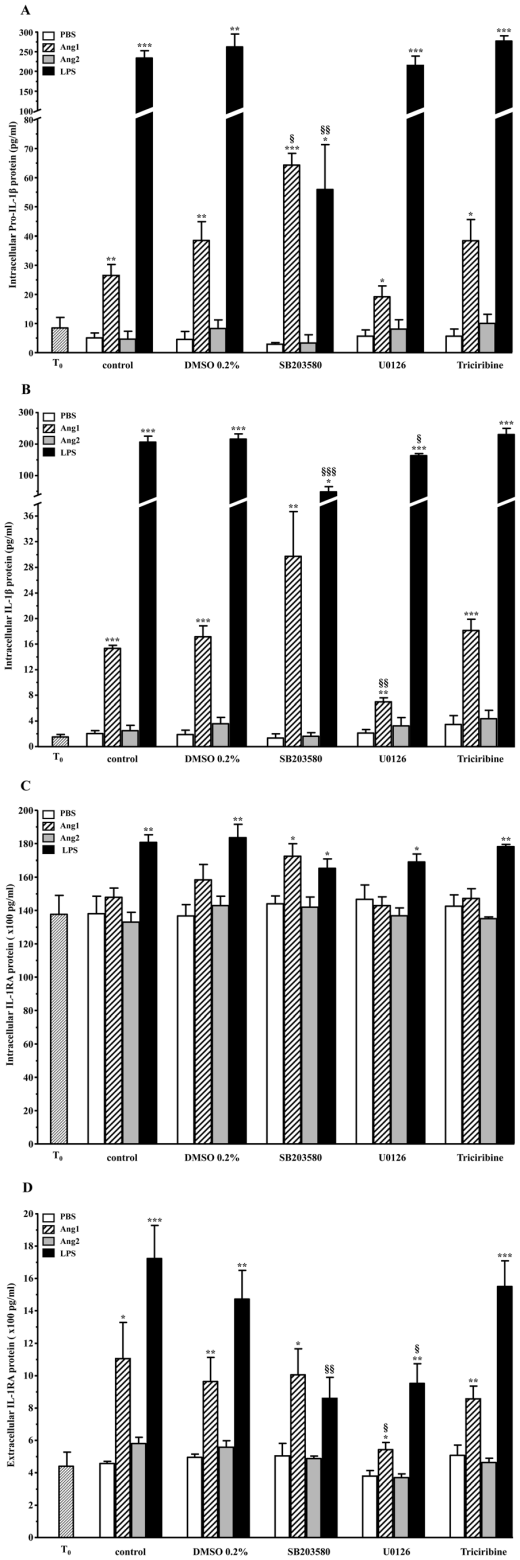
Effect of downstream signaling inhibitors on IL-1β and IL-1RA protein synthesis and release. Neutrophils were pretreated with inhibitors of Akt (Triciribine; 5 µM), p38 MAPK (SB203580; 10 µM), and p42/44 MAPKK (U0126; 20 µM), vehicle-DMSO (0.2%) or PBS for 30 minutes prior to a 2-hour agonist challenge. Concentrations of intracellular pro-IL-1β (**A**), IL-1β (**B**), IL-1RA (**C**) and released IL-1RA (**D**) were quantified by ELISA. Data are represented as mean ± SEM of at least three independent experiments. *p<0.05, **p<0.01, *** p<0.001 vs. PBS-control within each set (Dunnett's test); §p<0.05, §§p<0.01 and §§§p<0.001 vs corresponding agonist-DMSO (Tukey's test).

Basal (PBS) protein levels were not affected by the addition of DMSO or any of the inhibitors (Figure 7A–D). While p38 MAPK inhibition significantly increased Ang1-induced pro-IL-1β synthesis by 79%, from 38.5 pg/ml (Ang1-D) to 64.3 pg/ml (Ang1-SB), blockade of MEK1/2 lead to a 60% inhibition, with pro-IL-1β protein levels decreasing from 38.5 pg/ml (Ang1-D) to 19.2 pg/ml (Ang1-U0) (Figure 7A). As for LPS, blockade of p38 MAPK lead to a marked inhibition by 80% of pro-IL-1β protein expression, as levels dropped from 262.1 pg/ml (LPS-D) to 55.9 pg/ml (LPS-SB), despite similar treatment having no effect at the mRNA level. Blockade of MEK1/2 or Akt had no effect on LPS-driven pro-IL-1β levels (Figure 7A).

The inhibition pattern for mature IL-1β mimicked that of pro-IL-1β for both Ang1 and LPS (Figure 7B). Blockade of p38 MAPK increased Ang1-driven IL-1β protein levels by 86%, as levels jumped from 17.3 pg/ml (Ang1-D) to 29.9 pg/ml (Ang1-SB). Blockade of MEK1/2 lead to a 68% inhibition, as IL-1β levels decreased from 17.3 pg/ml (Ang1-D) to 7.1 pg/ml (Ang1-SB). In the case of LPS, as per the precursor protein, mature IL-1β levels were deeply affected by the blockade of p38 MAPK, witnessing a 77% effect inhibition as levels dropped from 219.2 pg/ml (LPS-D) to 52.0 pg/ml (LPS-SB). Surprisingly, the blockade of MEK1/2 activity had a partial but significant effect on IL-1β protein, corresponding to 24% inhibition, as levels went from 219.2 pg/ml (LPS-D) to 166.9 pg/ml (LPS-U0). For both Ang1 and LPS, the Akt pathway did not modulate IL-1β levels significantly. It should be noted that no pro- or mature IL-1β proteins were detected in the extracellular fraction, regardless of treatment (data not shown).

Intracellular IL-1RA protein levels were maintained between 13–18 ng/ml across treatments, with only a very slight increase and decrease in Ang1 and LPS-driven levels respectively, following p38 MAPK inhibition (Figure 7C). The lack of effect on the intracellular stores of IL-1RA protein following p38 MAPK blockade is noteworthy in the case of LPS, especially given the 63% reduction in the corresponding mRNA; these data demonstrate that the cell holds IL-1RA mRNA in large excess, and actually utilizes less than 40% of the total mRNA quantity it produces to convert into protein. However, the impact of inhibitors was immediately noticeable at the level of the release of IL-1RA (Figure 7D), suggesting that the neutrophil prioritizes of a having a constant pool of intracellular IL-1RA and will modify the amount released in response to different conditions. First, under Ang1, the dynamics of p38 MAPK-MEK1/2 mediation differed from those of IL-1β, in that only the blockade of MEK1/2 had a significant impact, equivalent to 65% inhibition, on extracellular IL-1RA levels, as levels dropped from 963.4 pg/ml (Ang1-D) to 542.5 pg/ml (Ang1-U0). Meanwhile, the blockade of p38 MAPK had no important impact on Ang1-mediated IL-1RA release (Ang1-SB), in line with the apparent lack in p38 MAPK contribution at the mRNA and the intracellular protein levels. For LPS, both the blockade of p38 MAPK and MEK1/2 exerted a negative effect on IL-1RA release: inhibition of p38 MAPK lead to a marked 75% inhibition, with levels dropping from 1 471.9 pg/ml (LPS-D) to 749.3 pg/ml (LPS-SB). Furthermore, blockade of MEK1/2 resulted in a marked 41% inhibition, with levels decreasing to 952.4 pg/ml (LPS-U0). Finally, for both Ang1 and LPS, the Akt pathway did not modulate IL-1RA release significantly.

## Discussion

Vessel destabilization, increase in permeability and leukocyte infiltration are hallmarks of both inflammation and angiogenesis. Under normal physiological conditions, these processes undergo a natural resolution or removal of inciting signals, a critical step in preventing disorganized vascular network formation and a sustained inflammatory reaction. During the resolution step, changes in the microenvironment through local mediators produced lead to an active “push-back” of infiltrating neutrophils, and serve to limit the activity of destabilizers such as VEGF, nitric oxide (NO) and Ang2 while increasing stabilizing elements such as Ang1 (resolution reviewed in [47];[48]). A previous study suggested that neutrophils might actually contribute to the resolution of inflammation based on their ability to produce endogenous anti-inflammatory mediators but little pro-inflammatory cytokines [23]. The present study supports those findings, as we show that during a 24-hour lifespan, neutrophils constitutively release endogenous anti-inflammatory mediator IL-1RA from a pool of stored protein that is continuously replenished, but no IL-1 agonists are produced or secreted. While we observed that Ang1 and LPS “prime” neutrophils to synthesize IL-1β *de novo*, in the absence of other signals, both precursor and mature IL-1β stores are retained within the intracellular compartment and are degraded over time. Additionally, the intracellular spikes in IL-1β levels were accompanied by parallel increases in the release of IL-1RA. Thus, we propose that neutrophils from healthy individuals naturally and intrinsically curtail the activity of IL-1 agonists, and “put the brakes” on the propagation of IL-1 mediated inflammation.

Of the 11 members of the IL-1 family of ligands, IL-1α and IL-1β are two major agonists with a demonstrated role in inflammation, angiogenesis, and hematopoiesis [49]. Both agonists bind to and activate IL-1 Receptor Type 1 (IL-1R1), and their activity is competitively antagonized by the endogenous IL-1RA. IL-1α and IL-1β are synthesized as precursor proteins; however, while IL-1α is active in both the precursor and the mature form upon release, IL-1β requires cleavage for activation and subsequent secretion. The importance of tight control over IL-1 production/processing is underlined by a number of serious inflammatory diseases, termed “autoinflammatory” (reviewed in [50]), that are closely correlated with deregulation in bioactive IL-1β secretion, and where the use of recombinant IL-1RA (anakinra) has clear therapeutic benefits. Details on the processing of IL-1β and its release are still unclear, but several groups have pointed to mechanistic cell-dependent differences. In monocytes, the IL-1β processing enzyme, caspase-1, is constitutively active, and mature IL-1β is released in large quantities (1 500 pg/ml) upon stimulation with LPS [39], [40] at a rate that is less than 20% of the total precursor pool [50]. For macrophages, an LPS challenge is insufficient; a second intracellular potassium (K^+^)-depleting stimulus is required to trigger the assembly of a complex called the inflammasome, the subsequent activation of caspase-1, and the processing and release of IL-1β [40], [49], [51], [52]. Based on our current data, we show that processing of pro-IL-1β in neutrophils is neither contingent on caspase-1 activation, nor on K^+^ depletion. Strictly speaking, K^+^ emptying did lead to mature IL-1β being detected outside the cells; however, these levels were the result of a simple externalization of already-accumulated mature IL-1β, with no active role *per se* for K^+^ depletion in the maturation step. These results provide evidence that IL-1β maturation in human neutrophils is distinct from the release process, and could be mediated by a mechanism other than caspase-1, as suggested by Greten *et al.* [36], or K^+^ efflux. The contribution of other components of the inflammasome to IL-1β processing in neutrophils, however improbable, should be considered and further explored.

### Intracellular mechanisms

Several studies have suggested that p38 MAPK regulates the synthesis and release of cytokines by many types of blood cells. For example, inhibition of the p38 MAPK pathway in monocytes, macrophages and neutrophils blocked LPS-induced protein transcription (including that of IL-1β), translation and subsequent cytokine release [53]–[57].

In general, the p38 MAPK pathway responds weakly to growth signals and is preferentially recruited by pro-inflammatory cytokines, whereas p42/44 MAPKs have been shown to be strongly activated by growth factors and growth-promoting hormones. Such is the case for Ang1 in mediating IL-8 *de novo* synthesis in neutrophils, a process that occurs through a p42/44 MAPK-dependent mechanism, and independently of p38 MAPK or Akt activity (56). Along the same lines, the present study suggests that p42/44 MAPK mediates most of the effects of Ang1 on IL-1 production in neutrophils. However, IL-1RA regulation appears to be less stringent than that of IL-1β: while antagonist *de novo* synthesis is not affected by any inhibition, and the release of IL-1RA is mostly regulated by a single signal transduction pathway (p42/44 MAPK), control of agonist production is two-fold involving not only p42/44 MAPK, but also p38 MAPK that exerts a negative regulatory role on the entirety of the IL-1β *de novo* synthesis process. The negative regulation exerted by p38 MAPK over the IL-1β synthesis process is likely a second insurance that IL-1β production remains tightly controlled when one of the two kinase pathways is unavailable, such as in the presence of a stronger pro-inflammatory signal.

A look into the downstream signaling governing the effects of LPS on IL-1 highlights differences that could be attributed to the potency of the inflammatory signal. According to our data, none of the studied pathways played a role in LPS-mediated IL-1β transcription, which is especially surprising for p38 MAPK given its similar role in macrophage cell lines [56]. However, p38 MAPK impacted both IL-1β translation and processing, as the precursor and the mature proteins were significantly down-regulated with p38 MAPK blockade. LPS also recruited p42/44 MAPK for IL-1β maturation, and both kinase pathways had a significant contribution to IL-1RA release. Thus, in the context of neutrophil IL-1 production, the recruitment of downstream signaling effectors is stimulus-dependent. Finally, because neutrophils maintained their constitutive synthesis of IL-1RA at the same level despite the inhibitors, it is likely that other signal transduction effectors mediate this process.

### Other potential targets of angiopoietin stimulation

qPCR arrays identified four additional potential targets of angiopoietin stimulation whose genetic changes could be rendered significant with more exhaustive kinetics studies: IL-8, CCR1 and Lymphotoxin B (LTB) for Ang1, and IL-8RB for Ang2. For instance, providing validation that the above targets might be significant is a recent finding that, when stimulated with Ang1 for 2 or more hours, neutrophils increased their IL-8 *de novo* synthesis[12], an effect that did not extend to Ang2. Lu P *et al.* demonstrated that IL-1α and IL-1β induced the production of a CCR1 ligand, CCL3, from human hepatomas [58]; while neither CCL3 nor any of the other CCR1 ligands (CCL4/MIP-1β and CCL5/RANTES) were affected by Ang1 treatment, it is possible that increases in IL-1β could drive CCR1 expression, increasing neutrophil responsiveness to surrounding tissue-derived corresponding ligands. As for Ang2, we have previously shown that it has similar agonistic capacity to Ang1 in mediating PAF synthesis, CD11b/CD18 activation and chemotaxis in neutrophils [4], [14], but this is the first time we report that Ang2 may modulate protein transcription. While the cross-talk between angiopoietins and the aforementioned proteins remains to be elucidated, the involvement of CCL3/CCR1 and IL-8RB in neutrophil migration could offer additional insight into the mechanisms governing differences in Ang1 and Ang2-driven neutrophil chemotaxis.

In conclusion, the identification of several inflammatory targets of angiopoietin stimulation provides further evidence of the implication of angiopoietins in acute inflammation. We showed that Ang1, a blocker of vessel permeability, induces transcription, translation and maturation of one pro-inflammatory IL-1 agonist, IL-1β. Perhaps to counter the damaging activities of IL-1β in the presence of a potential release signal, or perhaps to initiate resolution or to push back any additional neutrophil infiltration, neutrophils upregulate their release of IL-1RA in response to both Ang1 and the more potent pro-inflammatory signal LPS, as well as observed under TNF-α treatment [59]. These initial observations shed light on the complex interplay of inflammatory cells and mediators at the final stages of angiogenesis and acute inflammation, and provide a possible role for Ang1 in attenuating IL-1–related pathologies.
